# Characterizing Mental Health Status and Service Utilization in Chinese Americans With Type 2 Diabetes in New York City: Cross-Sectional Study

**DOI:** 10.2196/59121

**Published:** 2024-07-02

**Authors:** Yun Shi, Bei Wu, Nadia Islam, Mary Ann Sevick, Amanda J Shallcross, Natalie Levy, Kosuke Tamura, Han Bao, Ricki Lieu, Xinyi Xu, Yulin Jiang, Lu Hu

**Affiliations:** 1 Department of Population Health New York University Grossman School of Medicine New York University Langone Health New York, NY United States; 2 Center for Healthful Behavior Change New York University Grossman School of Medicine New York University Langone Health New York, NY United States; 3 New York University Rory Meyers College of Nursing New York, NY United States; 4 Department of Medicine New York University Grossman School of Medicine New York University Langone Health New York, NY United States; 5 Department of Wellness and Preventive Medicine Cleveland Clinic Cleveland, OH United States; 6 Socio-Spatial Determinants of Health Laboratory, Population and Community Health Sciences Branch, Division of Intramural Research National Institute on Minority Health and Health Disparities National Institutes of Health Bethesda, MD United States; 7 Jacobi Medical Center Bronx, NY United States; 8 Creighton University School of Medicine Omaha, NE United States

**Keywords:** mental health, diabetes distress, depression, anxiety, service utilization, psychological distress, type 2 diabetes, diabetes, United States, mental health burden, Chinese American, cross-sectional, telephone survey, stress, depressive symptoms, mental health care, mental health screening

## Abstract

**Background:**

Emerging evidence indicates that individuals with type 2 diabetes (T2D) are more prone to mental health issues than the general population; however, there is a significant lack of data concerning the mental health burden in Chinese Americans with T2D.

**Objective:**

The aim of this study was to explore the comorbid mental health status, health-seeking behaviors, and mental service utilization among Chinese Americans with T2D.

**Methods:**

A cross-sectional telephone survey was performed among 74 Chinese Americans with T2D in New York City. We used standardized questionnaires to assess mental health status and to gather data on mental health–seeking behaviors and service utilization. Descriptive statistics were applied for data analysis.

**Results:**

A total of 74 Chinese Americans with T2D completed the survey. Most participants (mean age 56, SD 10 years) identified as female (42/74, 57%), were born outside the United States (73/74, 99%), and had limited English proficiency (71/74, 96%). Despite nearly half of the participants (34/74, 46%) reporting at least one mental health concern (elevated stress, depressive symptoms, and/or anxiety), only 3% (2/74) were currently using mental health services. Common reasons for not seeking care included no perceived need, lack of information about Chinese-speaking providers, cost, and time constraints. The cultural and language competence of the provider was ranked as the top factor related to seeking mental health care.

**Conclusions:**

Chinese Americans with T2D experience relatively high comorbid mental health concerns yet have low service utilization. Clinicians may consider team-based care to incorporate mental health screening and identify strategies to provide culturally and linguistically concordant mental health services to engage Chinese Americans with T2D.

## Introduction

Type 2 diabetes (T2D) is a growing public health concern within the Chinese American community [1]. Recent epidemiological data have brought to light the startling prevalence of T2D among Chinese Americans, with 13.3% of this population diagnosed with T2D and an additional 33.8% classified as having prediabetes [2]. These statistics are especially worrisome considering that Chinese Americans constitute one of the fastest-growing immigrant populations in the United States, making up the largest Asian demographic with a population exceeding 5 million [3,4]. In comparison to their non-Hispanic White counterparts, Chinese Americans face distinct challenges with managing diabetes, including higher rates of poverty, limited English proficiency, and limited access to health care services [5,6]. This underscores the pressing need for research efforts to mitigate these disparities and improve diabetes management within the Chinese American community.

Accumulating evidence suggests that individuals with T2D have a higher likelihood of experiencing mental health issues such as depression, anxiety, and distress compared to those without T2D [7]. Among patients with T2D, those experiencing mental health issues are more likely to exhibit suboptimal adherence to diabetes self-care behaviors and poorer glycemic control compared to their counterparts without such issues [8,9]. Therefore, there are increasing calls from national organizations such as the American Diabetes Association and the Association of Diabetes Care and Education Specialists to screen for mental health issues and integrate mental health into diabetes care [10,11].

Cultural context significantly influences mental health–seeking behaviors and service utilization. For instance, in Chinese culture, mental health conditions are often stigmatized, leading to reluctance to discuss these issues or seek professional help [12]. This cultural barrier contributes to a significant gap in understanding the mental health burden among Chinese Americans with T2D, including their mental health status and health-seeking behaviors. The lack of data on these aspects hampers efforts to highlight the critical need for tailored mental health services in this underserved community, making it imperative to address these informational gaps to improve diabetes care outcomes.

Therefore, the aim of this study was to bridge critical knowledge gaps by assessing the comorbid mental health burden of Chinese Americans with T2D, including levels of depression, anxiety, general stress, and diabetes-specific stress. We also investigated both past and current health-seeking behaviors of these individuals, identified barriers to accessing mental health services, and evaluated preferences for mental health care. This report serves as an important first step to provide critical formative data to understand the mental health challenges faced by Chinese Americans with T2D and to inform the development of more effective, culturally sensitive interventions to address comorbid mental health and T2D burden in underserved communities.

## Methods

### Study Design

This cross-sectional survey was conducted as an ancillary study to a pilot randomized controlled trial testing the potential efficacy of a culturally tailored text messaging–based diabetes intervention to improve glycemic control in Chinese Americans with T2D [13]. The survey data were collected in New York City between October 2022 and January 2023. Standardized questionnaires were used to collect data on participants’ mental health status, mental health–seeking behaviors, and demographic information. Study materials were available in both English and Mandarin Chinese. A bilingual community health worker administered the surveys over the phone in the participant’s preferred language. To minimize participant burden, we opted for a phone-based survey over in-person interviews or online surveys. This approach is particularly well-suited for our target population of low-income, aging Chinese Americans with limited education and digital literacy, as phone surveys offer greater accessibility and inclusivity compared to the other two methods.

### Ethical Considerations

The study protocols were approved by the Institutional Review Board (IRB) of the New York University Grossman School of Medicine (s18-00609). Informed consent was obtained from all participants prior to their involvement in the study. During the call, a community health worker read the IRB-approved telephone consent script and obtained verbal consent. The entire process was recorded. The data were deidentified for analysis. Participants received a US $20 gift card after completing the survey.

### Participants

Participants were recruited from a registry comprising Chinese Americans with T2D who had previously taken part in diabetes studies led by the same study team. These earlier studies used community-based participatory research methods and partnered with several community-based organizations to evaluate mobile health interventions that were culturally and linguistically tailored to enhance glycemic control in low-income Chinese Americans with T2D [13-15]. The general inclusion criteria were as follows: (1) self-identified as Chinese American, (2) aged 18 years or older, (3) ability to speak and understand Mandarin or English, (4) self-reported or medical diagnosis of T2D, and (5) currently living in their homes and self-managing T2D at home. Exclusion criteria were individuals who were unable or unwilling to provide informed consent and those who had gestational diabetes. Further details regarding the inclusion and exclusion criteria for the study have been reported elsewhere [13,14].

### Measures

#### Mental Health Status

We used four standardized questionnaires to measure participants’ mental health status. The 9-item Patient Health Questionnaire (PHQ-9) [16], Hospital Anxiety and Depression Scale Anxiety Subscale (HADS-A) [17], Perceived Stress Scale (PSS) [18], and Diabetes Distress Scale (DDS) [19] were used to measure depressive symptoms, anxiety, general stress, and diabetes-specific distress, respectively. All these questionnaires are available in Chinese and have been validated among the Chinese population [20-23]. The PHQ-9 has a score range of 0 to 27, with higher scores indicating more severe depressive symptoms. A PHQ-9 score of 0-4, 5-9, 10-14, 15-19, or ≥20 indicates no, mild, moderate, moderately severe, or severe depression, respectively [24]. A score ≥10 indicates a significant risk for major depression [24]. The HADS-A has a score range of 0 to 21, with higher scores indicating more severe anxiety symptoms. A score of 0-7, 8-10, or ≥11 on the HADS-A scale indicates normal, borderline abnormal, or abnormal anxiety, respectively [17]. The PSS has a score range of 0 to 40, with higher scores indicating higher perceived stress. A score of 0-13, 14-26, or ≥27 indicates low, moderate, or high stress levels, respectively [25]. The DDS has a score range of 1 to 6, with higher scores indicating higher diabetes-specific distress. A total score ≥2 indicates a moderate or higher distress level and is considered clinically important [26].

#### Mental Health–Seeking Behavior

We used an investigator-developed questionnaire to collect information regarding participants’ mental health service use and health-seeking behavior, including whether they had a mental health–related diagnosis, whether they had seen a mental health provider in the past 12 months, whether they were currently seeking mental health services, reasons for not seeing a provider, coping strategies for stress, preferences for mental health services, and factors to consider when seeking mental health services.

### Statistical Analysis

We performed descriptive analyses for all variables, reporting frequencies and percentages for categorical variables and means with SDs for continuous variables. To evaluate the internal consistency of the standardized questionnaires, we computed Cronbach α coefficients. We used R version 4.2.1 [27] for data analysis.

## Results

### Demographic Characteristics of Participants

Data from 74 Chinese Americans with T2D were analyzed in this study. As shown in Table 1, most participants (mean age 56, SD 10 years) identified as female (42/74, 57%), were born outside the United States (73/74, 99%), were currently married (57/74, 77%), had a high school education or less (56/74, 76%), and had a household annual income <US $25,000 (49/74, 66%). Approximately 18% (13/74) of the participants were unemployed and 58% (43/74) had Medicaid health coverage. On average, participants have been residing in the United States for 18 (SD 11) years, although 96% (71/74) of them had limited English proficiency.

**Table 1:** Demographic characteristics of participants (N=74).

Characteristics		Value
Age (years), mean (SD)^a^		56 (10)
Female sex, n (%)		42 (57)
Currently married or cohabitating, n (%)		57 (77)
**Education level, n (%)**		
	Less than high school	29 (39)
	High school graduate	27 (37)
	More than high school	18 (24)
**Annual income (US $), n (%)**		
	<25,000	49 (66)
	≥25,000	23 (31)
	Declined to answer or don’t know	2 (3)
**Employment status, n (%)**		
	Employed	45 (61)
	Not employed, not working	13 (17)
	Retired	16 (22)
**Insurance type, n (%)^b^**		
	Private insurance	7 (9)
	Medicaid	43 (58)
	Medicare	16 (22)
	Other types of public/government insurance	15 (20)
	No insurance	6 (8)
Born outside United States, n (%)		73 (99)
Limited English proficiency, n (%)		71 (96)
Duration of US residency (years), mean (SD)^c^		18 (11)

^a^Owing to missing values, the sample size is 73.

^b^Participants were asked to select all insurance types they possessed (ie, “check all that apply”).

^c^Owing to missing values, the sample size is 72.

### Mental Health Status

Table 2 displays the mental health status of the participants. Among the 74 participants in the study, 46% (34/74) had at least one mental health concern, which included 9% (7/74) with a diagnosis of either major depression or anxiety disorders before study entry and an additional 36% (27/74) with either elevated stress, depressive symptoms, or anxiety. Specifically, 27% (20/74) had moderate or higher levels of general stress, 26% (15/57) had moderate or higher levels of diabetes-specific distress, 9% (7/74) reported mild or higher levels of depressive symptoms, and 7% (5/74) had borderline or abnormal anxiety.

**Table 2:** Mental health status of the participants (N=74).

Mental health status		Participants, n (%)	Potential score range	Cronbach α
**Self-reported mental health-related diagnosis, n (%)**			—^a^	—
	No	67 (91)	—	
	Yes (depression or anxiety)	7 (9)	—	
	Major depression	4 (5)	—	
	Anxiety disorders	3 (4)	—	
**Depressive symptoms (PHQ-9^b^ level), n (%)**			0-27	0.82
	No	67 (91)	0-4	
	Mild	4 (5)	5-9	
	Moderate	2 (3)	10-14	
	Moderate-severe	1 (1)	15-19	
	Severe	0 (0)	20-27	
**Anxiety (HADS-A^c^ level), n (%)**			0-21	0.79
	Normal	69 (93)	0-7	
	Borderline abnormal	3 (4)	8-10	
	Abnormal	2 (3)	11-21	
**Perceived stress (PSS^d^ level), n (%)**			0-40	0.56
	Low	54 (73)	0-13	
	Moderate	19 (26)	14-26	
	High	1 (1)	27-40	
**Diabetes distress (DDS^e^ level), n (%)^f^**			1-6	0.92
	Moderate to higher distress	15 (26)	2-6	
	Low distress	42 (74)	1	

^a^Not applicable.

^b^PHQ-9: 9-item Patient Health Questionnaire.

^c^HADS-A: Hospital Anxiety and Depression Scale Anxiety Subscale.

^d^PSS: Perceived Stress Scale.

^e^DDS: Diabetes Distress Scale.

^f^The sample size for diabetes distress is 57.

### Mental Health–Seeking Behaviors

As shown in Table 3, only 7% (5/74) of the participants reported ever having seen a mental health professional and 3% (2/74) were currently seeking mental health services. Not seeking mental health services was attributed to no perceived need (69/74, 93%), lack of information about Chinese-speaking providers (3/74, 4%), cost (1/74, 1%), and time constraints (1/74, 1%). Participants managed stress through various means such as listening to music/watching TV (52/74, 70%), engaging in physical activities (20/74, 27%), or talking to family and friends (26/74, 35%). Concerning preferences for the format of mental health services, most participants favored individual sessions (28/74, 38%) and in-person consultations (32/74, 43%). The three primary factors participants considered when seeking mental health services were the cultural and language competence of the provider (26/74, 35%), confidentiality (16/74, 22%), and cost (13/74, 18%).

**Table 3:** Health-seeking behaviors (N=74).

Health-seeking behaviors			Participants, n (%)
Have seen a provider in the past 12 months			5 (7)
**Currently seeking mental health services**			2 (3)
	Talk to a therapist	1 (1)	
	See a psychiatrist	1 (1)	
	Taking medication	1 (1)	
**Reasons for not seeking mental health services**			
	No need	69 (93)	
	Lack of information about Chinese-speaking providers	3 (4)	
	Cost	1 (1)	
	Don’t have time	1 (1)	
	Stigma	0 (0)	
	Transportation barriers	0 (0)	
**Strategies to cope with stress**			
	Listen to music/watch TV/entertainment	52 (70)	
	Talk to family/friends	26 (35)	
	Physical activity/exercise	20 (27)	
	No stress	5 (7)	
	Drinking	2 (3)	
	Seek help from mental health providers	1 (1)	
	Meditation	1 (1)	
	Smoking	1 (1)	
	Binge eating	1 (1)	
	Keep silence	1 (1)	
	Sleep	1 (1)	
**Mental health services preference**			
	Individual	28 (38)	
	Group	9 (12)	
	Neither. I don’t want to use mental health services	25 (34)	
	Either	12 (16)	
**Mental health services format preference**			
	In-person	32 (43)	
	Don’t want to use mental health services	20 (27)	
	Phone	12 (16)	
	Any format	8 (11)	
	Video-based	2 (3)	
**Factors to consider when seeking mental health services**			
	A provider that understands my language/culture	26 (35)	
	Confidentiality	16 (22)	
	Cost	13 (18)	
	Access	7 (9)	
	Close to home	5 (7)	
	Flexibility	4 (5)	
	Others	10 (14)	
	No need	8 (11)	
	Qualified doctor	1 (1)	
	No idea	1 (1)	

## Discussion

### Principal Findings

To the best of our knowledge, this is the first study to examine the comorbid mental health status and health-seeking behaviors among Chinese Americans with T2D. Nearly half (34/74, 46%) of the participants had mental health concerns. However, only 7% (5/74) have previously sought help from a mental health professional, while 3% (2/74) were using mental health services. The major reasons for not seeking mental health services were no perceived need (69/74, 93%), lack of information about Chinese-speaking providers (3/74, 4%), cost (1/74, 1%), and time constraints (1/74, 1%).

Our results indicate a high prevalence of mental health issues among Chinese Americans with T2D. This prevalence rate is higher than the rates reported in another study; according to a secondary data analysis using information from the 2019 US Medical Expenditure Panel Survey, the rates of mental health burden were 25.7% in White participants, 14.9% in Black participants, 12.8% in Hispanic participants, and 10.2% in Asian/Native Hawaiian participants [28]. Our higher percentage may be due to the fact that the study based on national data focused only on self-report diagnoses of mental health issues, whereas our study included self-reported diagnoses plus the screening results of elevated levels of depressive symptoms, anxiety, general stress, and diabetes-specific stress. Notably, our findings based on self-reported mental health–related diagnoses (7/74, 9%) align closely with the findings from national data (10.2%).

Despite a relatively high mental health burden, our participants reported a low use rate and perceived no need to seek mental health services. These findings align with previous studies reporting that Chinese Americans may view mental health symptoms as negative emotions rather than a disorder and are thus less likely to seek professional help [29]. This could be attributed to traditional Chinese values of viewing negative emotions as “part of life,” accepting destiny, and withstanding hardship [30]. These cultural influences can contribute to the underrecognition of mental health issues and further prevent health-seeking behaviors.

Other studies have previously identified stigma as a significant contributor to the underutilization of mental health services among Chinese adults [12]. Surprisingly, none of our participants reported stigma as a barrier to mental health–seeking. This finding could be related to social desirability bias, where participants might deny their stigmatized perceptions of mental health services to conform to societal expectations. In subsequent focus group discussions, participants brought up the stigma surrounding mental health within Chinese communities. Another piece of evidence that supports why our patients did not report stigma as a barrier is our finding that participants ranked confidentiality, a factor often associated with stigma, as the second most important consideration in seeking mental health services. This emphasis on confidentiality suggests an underlying concern, indicating that although participants may not have reported stigma, their fear of mental health service usage disclosure may serve as a significant stigma-related barrier to mental health service access.

Additionally, our participants indicated a key barrier to seeking mental health services: the lack of information about providers proficient in their language and cultural context. This finding is largely because Chinese Americans do not feel comfortable speaking to a mental health professional who does not share the same cultural background or speak the same language [31]. This highlights a gap in the system where the dearth of culturally competent providers leads to a reluctance to seek necessary mental health services. Indeed, our findings reveal that the presence of a culturally and linguistically competent provider is the most frequently attributed factor Chinese Americans with T2D consider when seeking mental health services. Consequently, these findings emphasize the urgent need to embrace cultural competency and intervention adaptation to meet the unique needs of diverse populations.

### Strengths and Limitations

To our knowledge, this study is the first to describe the comorbid mental health burden and health-seeking behaviors in Chinese Americans with T2D. By addressing this significant gap, our study lays the foundation for future investigations and interventions that target the mental health and diabetes management needs of this particular minoritized group. In addition, we used a community-based participatory research design and included many low-income Chinese Americans with limited English proficiency, who are often understudied and not included in large national data sets due to language barriers. This study thus provides critical preliminary data on this underserved population.

There were several limitations of our study. First, the sample size was relatively small with all participants recruited from New York City, which may limit the generalizability of our findings to the broader Chinese American community. Factors such as access to Chinese-speaking health care providers and community support may vary significantly across different regions of the United States. Furthermore, the participants of this study had previously taken part in our prior research studies, potentially biasing our sample toward individuals more inclined to seek care. This suggests that the mental health status and underutilization of mental health services could be even more pronounced in the broader population. While two of our recent studies used electronic medical records to confirm the T2D diagnosis [14,15], one of our earlier studies relied on a self-reported diagnosis of T2D [13]. In addition, our study relied on self-report measures and there may be inherent biases and limitations associated with self-reported data, such as social desirability bias or recall bias. For example, participants may conceal their previous diagnosis or mental health service use due to stigma and/or feelings of shame. Future studies could incorporate electronic medical records or use multiple sources of data to overcome these limitations to provide a more comprehensive understanding of the mental health status and health-seeking behaviors among Chinese Americans with T2D.

### Conclusions

Mental health issues among Chinese Americans with T2D are prevalent, yet their utilization of mental health services remains strikingly low. This discrepancy can be attributed to several factors, including the low perceived need for mental health support and the lack of knowledge of culturally and linguistically competent providers. To address this issue, it is imperative to incorporate mental health screening into comprehensive T2D care and raise awareness of mental health issues within this population. Moreover, the development and implementation of culturally tailored interventions are crucial to effectively reach and engage Chinese Americans in mental health treatment.

## Abbreviations

DDS: Diabetes Distress Scale
HADS-A: Hospital Anxiety and Depression Scale Anxiety Subscale
IRB: Institutional Review Board
PHQ: Patient Health Questionnaire
PSS: Perceived Stress Scale
T2D: type 2 diabetes

## Data Availability

The data presented in this study are available on request from the corresponding author.
